# Editorial: Active methodologies: exploring the impact on motivation and psychological variables in physical education

**DOI:** 10.3389/fpsyg.2024.1476430

**Published:** 2024-10-07

**Authors:** Alfonso Valero-Valenzuela, Luz Amelia Hoyos Cuartas, Diego Andrés Heredia-León, Patxi León-Guereño

**Affiliations:** ^1^SAFE Research Group, Sport Sciences Faculty, University of Murcia, San Javier Campus, Murcia, Spain; ^2^Docente de Planta Universidad Pedagógica Nacional, Bogotá, Colombia; ^3^Carrera de Pedagogía de la Actividad Física y Deporte, Universidad Católica de Cuenca, Cuenca, Ecuador; ^4^Department of Physical Activity and Sport Science, Faculty of Education and Sport, University of Deusto, Donostia-San Sebastian, Gipuzkoa, Spain

**Keywords:** sport pedagogy, students, pedagogical models, physical activity, active learning, motivation

Physical Education, like the educational system in general, has tried to adapt to the new generations of students; to this end, numerous changes have been proposed at the methodological level. Unlike conventional teaching methods, where the teacher takes the leading role, active methodologies emphasize student's continuous involvement. This transformative shift in educational approaches, centered on dynamic teaching strategies and active methodologies, seeks to respond to the new educational context. These methods encompass various elements such as teaching games for understanding, sports education, gamification, or cooperative learning, which have all meant a major change in perspective and focus for physical education in recent decades.

Active methodologies aim to place students at the forefront of the classroom experience, making them the primary agents of their own learning. This approach engages students in activities, decision-making processes, and self-evaluation. Thus, a significant focus has been placed on identifying teaching methods that foster largely self-driven motivation in students. This approach aims to enhance physical literacy, enhance factors associated with self-esteem and self-perception, fulfill essential psychological needs, improve social dynamics and interpersonal connections, and encourage a lasting commitment to physical activity. However, despite the attributed benefits, the implementation of these methodologies, as well as their effects on the students, have not been adequately analyzed. Consequently, the objective of this compilation has been to consolidate studies examining the application of dynamic teaching approaches in physical education settings and the resultant effects of their adoption.

This Research Topic collects investigations related to different areas of focus like the effect of online classes on sleep, physical activity, and cognition functioning of physical education students, the analysis of experienced coaches and physical education teachers' teaching methods and their perception, teachers' perception and students' perspectives related to activities modalities, as well as instructional settings during primary school physical education classes. Furthermore, researchers examined the validity of the Chinese adaptation of the exercise empowerment scale when applied to college students. Additionally, they investigated how the Sport Education model influenced student motivation, comparing results between Kuwaiti and American students.

The Haddad et al. study investigates the impact of online vs. face-to-face learning environments on sleep quality, physical activity, and cognitive functioning among physical education students. The research combines wrist actigraphy, the Pittsburgh Sleep Quality Index, and the Cambridge Neuropsychological Test Automated Battery. A study involving 19 students was conducted over a 4-week period, alternating between online and in-person class formats. The findings indicated that there were no notable distinctions in cognitive performance or sleep quality when comparing the two learning environments. However, findings showed significant differences in Paired Associates Learning and weekday step counts in the face-to-face setting. The findings indicate that virtual educational settings likely do not negatively influence sleep patterns or overall cognitive abilities. However, they may have some effect on particular physical activity levels and certain mental tasks.

The study by Demiral and Nazıroǧlu seeks to explore how coaches and physical education teachers perceive the application and significance of various teaching methods. Two assessment tools, namely the “Coaches' Instructional Methods Utilization Scale” and the “Physical Education Teachers' Perception of Teaching Methods Scale,” were employed in a study involving 114 coaches and 115 physical education teachers. These participants, who volunteered for the research, were drawn from three randomly chosen provinces in Türkiye. The Cronbach Alpha values ranged between 0.89 and 0.93 for the “Coaches' Instructional Methods Utilization Scale” and between 0.90 and 0.96 for the “Physical Education Teachers' Perception of Teaching”. A notable correlation was found in the techniques employed by both coaches and physical education instructors. Additionally, their understanding and views regarding these methodologies showed significant parallels. The study revealed gender-based disparities in the perception and application of teaching methodologies among physical education instructors. Notably, female educators in this field demonstrated a lower appreciation for various teaching approaches. Furthermore, the research uncovered significant variations in the teaching methods employed by physical education teachers, highlighting a notable difference in pedagogical practices across genders.

The Yan et al. study sets out to understand the association between teaching practices, teacher confidence, competence, self-efficacy, and the resulting student outcomes. Researchers gathered information through video recordings, which were subsequently analyzed using the MASTER Observation Tool. Additionally, questionnaires were employed to collect data on participants' demographic details, self-assessed teaching confidence and competence, self-efficacy, and student outcomes. The researchers employed a one-way analysis of variance (ANOVA) to examine the relationships between various teacher attributes and the outcomes observed in both teachers and students. This investigation encompassed a diverse sample, including ten elementary schools, with participation from 597 pupils and 16 physical education instructors. The research at hand substantiated that educators allocated a significant portion of physical education class time to training-oriented exercises, with instructional periods coming in second. The study also revealed that the setting of the class and the number of students present influenced both teaching methods and student performance. However, gender did not appear to be a determining factor in these aspects. The study contributes to our understanding of PE instruction in Chinese primary schools and offers preliminary evidence to improve future PE teaching strategies.

To address the degree of exercise empowerment, The Exercise Empowerment Scale (EES) has been formulated. The research conducted by Weng et al. in 2024 focuses on creating and validating a Chinese adaptation of the EES. This study involved a substantial sample of 585 Chinese university students. The findings of the research suggest that the EES-C demonstrates strong and reliable statistical characteristics, making it an appropriate tool for use with Chinese university students. The modified EES-C serves as a foundation for investigating the factors that predict physical activity among Chinese participants. Future research should aim to confirm whether these findings can be applied to other groups.

The study by Albaloul et al. aims to assess the effects of Sport Education (SE) on the motivation of Kuwaiti students, examine any differing impacts of SE on the motivation of Kuwaiti vs. American students, and investigate students' perceptions of SE in both countries. The study employed a replicated mixed-methods design with quasi-experimental pre-test and post-test components. The research involved two distinct groups of secondary school students: 33 boys and girls from two classes in the southwestern United States and 37 students from two classes in Kuwait. To analyze the data, researchers utilized repeated measures and mixed ANOVAs, followed by *t*-tests for further examination. Qualitative data were analyzed using a thematic analysis technique. The findings indicate that Kuwaiti students in the group taught exclusively with SE experienced significant improvements in their perceived interest/enjoyment, perceived competence, effort/importance, and pressure/tension scores. In summary, social engagement (SE) has proven to be effective in enhancing the motivation of Kuwaiti students in physical education. This motivating impact of SE has been consistently observed among both Kuwaiti and American students.

In conclusion, the studies published in this Research Topic collect Physical Education-related manuscripts developed in different countries such as China, Türkiye, Kuwait, and America. Within these investigations, Physical Education teachers' perceptions, the effect of the Sport Education Model on students' motivation, and a validation of the EES were developed. Moreover, the impact of online classes on PE students' sleep, cognition, and physical activity was also analyzed.

